# Cerebrospinal Fluid Findings in Patients With Autoimmune Encephalitis—A Systematic Analysis

**DOI:** 10.3389/fneur.2019.00804

**Published:** 2019-07-25

**Authors:** Tetyana Blinder, Jan Lewerenz

**Affiliations:** Department of Neurology, Ulm University, Ulm, Germany

**Keywords:** autoimmune encephalitis, antineuronal antibodies, cerebrospinal fluid, pleocytosis, oligoclonal bands, NMDAR antibodies, LGI1 antibodies, GAD antibodies

## Abstract

Autoimmune encephalitides (AIE) comprise a group of inflammatory diseases of the central nervous system (CNS), which can be further characterized by the presence of different antineuronal antibodies. Recently, a clinical approach for diagnostic criteria for the suspected diagnosis of AIE as well as definitive AIE were proposed. These are intended to guide physicians when to order the antineuronal antibody testing and/or facilitate early diagnosis even prior to the availability of the specific disease-confirming test results to facilitate prompt treatment. These diagnostic criteria also include the results of basic cerebrospinal fluid (CSF) analysis. However, the different antibody-defined AIE subtypes might be highly distinct with regard to their immune pathophysiology, e.g., the pre-dominance of specific IgG subclasses, IgG1, or IgG4, or frequency of paraneoplastic compared to idiopathic origin. Thus, it is conceivable that the results of basic CSF analysis might also be very different. However, this has not been explored systematically. Here, we systematically reviewed the literature about the 10 most important AIE subtypes, AIE with antibodies against NMDA, AMPA, glycine, GABA_A_, and GABA_B_ receptors as well as DPPX, CASPR2, LGI1, IgLON5, or glutamate decarboxylase (GAD), with respect to the reported basic CSF findings comprising CSF leukocyte count, total protein, and the presence of oligoclonal bands (OCB) restricted to the CSF as a sensitive measure for intrathecal IgG synthesis. Our results indicate that these basic CSF findings are profoundly different among the 10 different AIE subtypes. Whereas, AIEs with antibodies against NMDA, GABA_B_, and AMPA receptors as well as DPPX show rather frequent inflammatory CSF changes, in AIEs with either CASPR2, LGI1, GABA_A_, or glycine receptor antibodies CSF findings were mostly normal. Two subtypes, AIEs defined by either GAD, or IgLON5 antibodies, did not fit into this general pattern. In AIE with GAD antibodies, positive OCBs in the absence of other changes were typical, while the CSF in IgLON5 antibody-positive AIE was characterized by elevated protein.

## Introduction

Autoimmune encephalitides (AIE) are inflammatory diseases of the central nervous system (CNS) (1). As a differential diagnosis for infectious encephalitides, epilepsy of other causes, or cognitive deterioration of non-inflammatory origin, the diagnosis of AIE is often established by the detection of subtype-specific antibodies against different neuronal surface antigens in the cerebrospinal fluid (CSF), the blood or both. It has been suggested that rapid immunosuppressive treatment improves the outcome of patients with AIE (2). However, the specific antibody testing usually takes several days. Thus, CSF findings like CSF pleocytosis, increased protein, and the presence of oligoclonal bands (OCB) restricted to the CSF might prove an inflammatory origin of neurological disturbances compatible with an AIE prior to the specific test results, thereby supporting the diagnosis and triggering early treatment.

Recently, diagnostic criteria for AIEs were proposed, which also incorporate CSF findings (3). Of note, only CSF pleocytosis was chosen as supporting findings for the diagnostic categories of possible AIE or definitive limbic encephalitis of autoimmune origin. In contrast, for the diagnostic category of possible NMDAR encephalitis, both positive OCB and pleocytosis were considered as supportive CSF findings. Of note, it has been reported that inflammatory CSF changes, although common in patients with NMDAR encephalitis (4), might be rare in other AIE subtypes, e.g., AIE associated with LGI1 antibodies (5, 6). Thus, the likelihood that inflammatory CSF findings support the suspected AIE diagnosis might strongly depend on the underlying disease subtype in an individual patient. However, this relationship has not been studied systematically.

In this analysis, we systematically reviewed the literature regarding 10 AIE subtypes with well-defined antibodies. We extracted the reported CSF findings both on the basis of group findings as well as of data reported for individual patients whenever possible. In these different data sets, we analyzed the cumulative reported frequencies and levels of CSF pleocytosis, elevated total protein as well as the frequency of positive OCB with respect to the antibody-defined specific AIE subtype. In addition, we analyzed the typical combination of the three basic values when reported for individual patients to characterize the typical CSF result pattern in the 10 AIE subtypes.

## Methods

We reviewed the published literature using the PubMed data base (https://www.ncbi.nlm.nih.gov/pubmed) of the National Center for Biotechnology Information for publications published until December 31st, 2018 with regard to AIE with AMPA receptor (AMPAR), CASPR2, DPPX, glutamate decarboxylase (GAD), glycine receptor (GlyR), IgLON5, GABA_B_ receptor (GABA_B_R), GABA_A_ receptor (GABA_A_R), LGI1, and NMDA receptor (NMDAR) antibodies using the following search terms: “NMDA,” “AMPA,” “CASPR2,” “DPPX,” “GAD,” “GlyR,” “IgLON5,” “GABA_B_R,” “GABA_A_R,” or “LGI1” in combination with “encephalitis,” “IgLON5” combined with “case report,” “CASPR2” in combination with “seizure.” The hits were critically reviewed and all publications that reported CSF findings for two or more patients for one of the 10 specific antibodies were selected for further analysis. However, in AIE subtypes for which this strategy identified very few patients (<15), namely AIEs with CASPR2, DPPX, GABA_B_R, and GABA_A_R antibodies, for individual CSF data comprising all three parameters, we also incorporated data from reports of two or one patients. It was carefully checked whether some patients might be reported in more than one publication. These patients were only included once. As characterizing the CSF abnormalities in children was beyond the scope of this paper, patients younger than 13 years were excluded.

Basically, two types of data were extracted from the selected publications, group data and individual data. Most often, only group data were reported, e.g., percentage of patients with pleocytosis among all with information about CSF cell count available. For female gender, CSF pleocytosis, increased protein as well as the presence of OCB, the percentage of patients positive for one of these findings was calculated as follows: For each specific AIE subtype, the total number of patients positive for one of these findings was obtained by adding up all these patients reported in all selected publications. These were compared to the total number of patients for whom information regarding this parameter could be extracted from these publications. If one CSF parameter was reported as either normal or abnormal for some patients but no information was given for other patients, we assumed that the results of this analysis were not available for the latter. Thus, these patients were not counted for the total number of patients for this parameter. If only pathological values were specifically reported, it was assumed that the data were available but normal in all other patients unless it was specifically mentioned that these values were not available. Of note, the normal values for CSF cell count varied from up to 4 to 5 cells/μl among the publications. In addition, the upper normal limit for total protein ranged from 350 to 500 mg/l. When reported, we extracted the exact results for the CSF cell count as cells/μl and CSF total protein as mg/l and regarded cell counts up to 4 cells/μl and protein levels up to 450 mg/l used for majority of publications applied these cut-offs. Median, minimum, maximum, and interquartile range were calculated using the Graph Prism Software.

## Results

**Figure 1:**
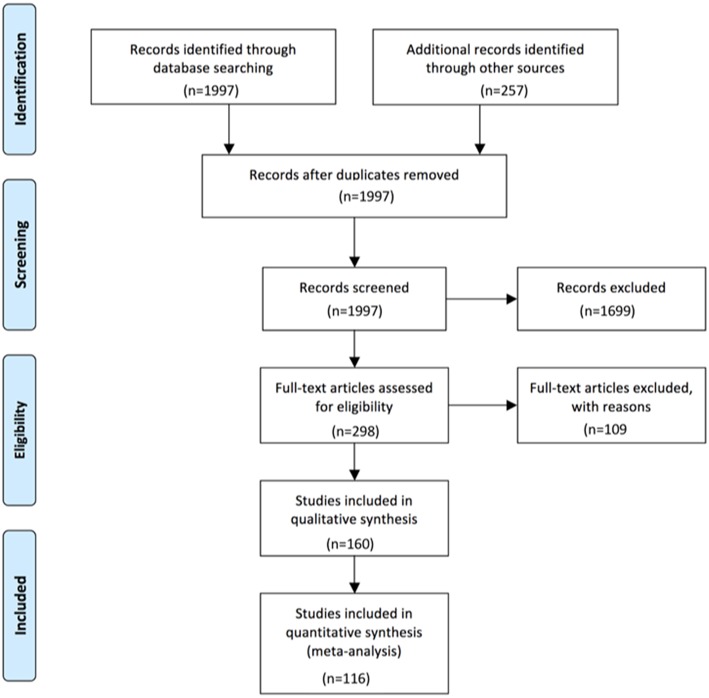
Study profile.

For all antibody-defined AIE subgroups combined, we could identify 116 publications that matched our search criteria and contained relevant information for the intended analyses. Ten were identified for AIE with AMPAR antibodies (7–16), 15 for CASPR2 (16–30), eight for DPPX (31–38), four for GABA_A_R (15, 39–41) with patients reported by the Pettingil et al. being excluded as these were not tested for GABA_A_R antibodies in CSF and represented a different phenotype (42), 15 for GABA_B_R antibodies (12, 15, 16, 43–54), 15 for GAD antibodies (19, 22, 55–67), five for GlyR antibodies (68–72), 14 for IgLON5 antibodies (73–86), 25 for LGI1 antibodies (5, 6, 15, 16, 19–22, 30, 52, 77, 87–100), and finally 27 for NMDAR antibodies (4, 15, 16, 19, 22, 52, 62, 88, 89, 101–118). The history of the data search and the results are depicted in Figure 1. With regard to the group data, information regarding the presence or absence of CSF pleocytosis was available for 1,305 patients total, while less information was available for increased CSF protein and presence of OCB with a total of 1,001 and 610 patients, respectively (Table 1). The data set for AIE with DPPX antibodies was the smallest with 29, 16, and 19 patients for percentages of either pleocytosis, increased protein or presence OCB, respectively, while the number of reported CSF findings was highest for AIE patients with NMDAR antibodies. Here, the frequency of pleocytosis was reported in 532 patients, the occurrence of increased protein in 433, and the presence of OCB in 196 patients. However, this kind of group data did neither allow to analyze the typical age of onset, CSF cell count or CSF protein levels nor the frequency of co-occurrence of pleocytosis, increased protein and presence of OCB in individual patients. Thus, whenever reported individually, we also extracted the age, gender, CSF cell count in cells per μl, total protein as mg/l and presence or absence of OCB for each patient. However, the size of the groups of patients with individual data available was substantially lower compared to the cohorts for which group data were combined (Table 1, Supplementary Table 1). The group size for each antibody-defined AIE was smallest for individual patients with information regarding all three basic CSF parameters whether normal of pathological available, ranging from 5 to 34 for DPPX- and GAD-antibody associated AIE, respectively (Table 1). In general, the proportion of patients with individual values compared to group data for gender, cells, CSF protein, and OCB was lowest for patients with NMDAR, GAD, GABA_A_R, and again NMDAR antibodies with 12, 8, 9, and 18%, respectively (Supplementary Table 1). In contrast, for gender and AMPAR and GABA_A_R antibodies and OCB and GABA_A_R antibodies all individuals identified had individual data. When the frequencies of CSF pleocytosis, elevated CSF protein, or positive OCB in the cohorts with individual exact values was compared to the those generated by adding up group data, we found that for 6 of the 10 well-defined antibodies the percentage of either pathological CSF cell count and elevated protein values was significantly higher in patients with individual exact values given compared to the group data. This indicates a strong bias for over-reporting of pathological in comparison to normal values (Supplementary Table 2).

**Table 1:** Number of patients with AIE with disease-specific antibodies identified by the literature search either as grouped data, with individual exact data or all three major parameters available.

**Anti- body target**	**Group data**					**Individual exact data**					**Cells + TP + OCB**
	**Age**	**Gender**	**Cells**	**TP**	**OCB**	**Age**	**Gender**	**Cells**	**TP**	**OCB**	
AMPAR	51	50	51	43	19	50	50	34	18	16	15
CASPR2	103	100	91	59	38	29	22	17	8	8	6
DPPX	39	39	29	16	19	19	19	15	5	18	5
GABA_A_R	24	24	24	22	24	24	24	10	2	24	8
GABA_B_R	130	130	112	72	19	125	124	79	46	18	8
GAD	192	192	76	76	163	65	65	6	12	65	34
GlyR	77	77	62	62	62	32	32	11	11	18	18
IgLON5	52	52	37	36	29	20	20	4	9	16	14
LGI1	351	348	291	182	41	112	109	30	25	33	25
NMDAR	486	504	532	433	196	58	60	65	42	35	21
Sum	1,505	1,516	1,305	1,001	610	534	525	270	178	251	154

*TP, total protein; OCB, oligoclonal IgG restricted to the CSF; Cells+TP+OCB, Data for CSF cells, total protein as well as OCB*.

**Figure 2:**
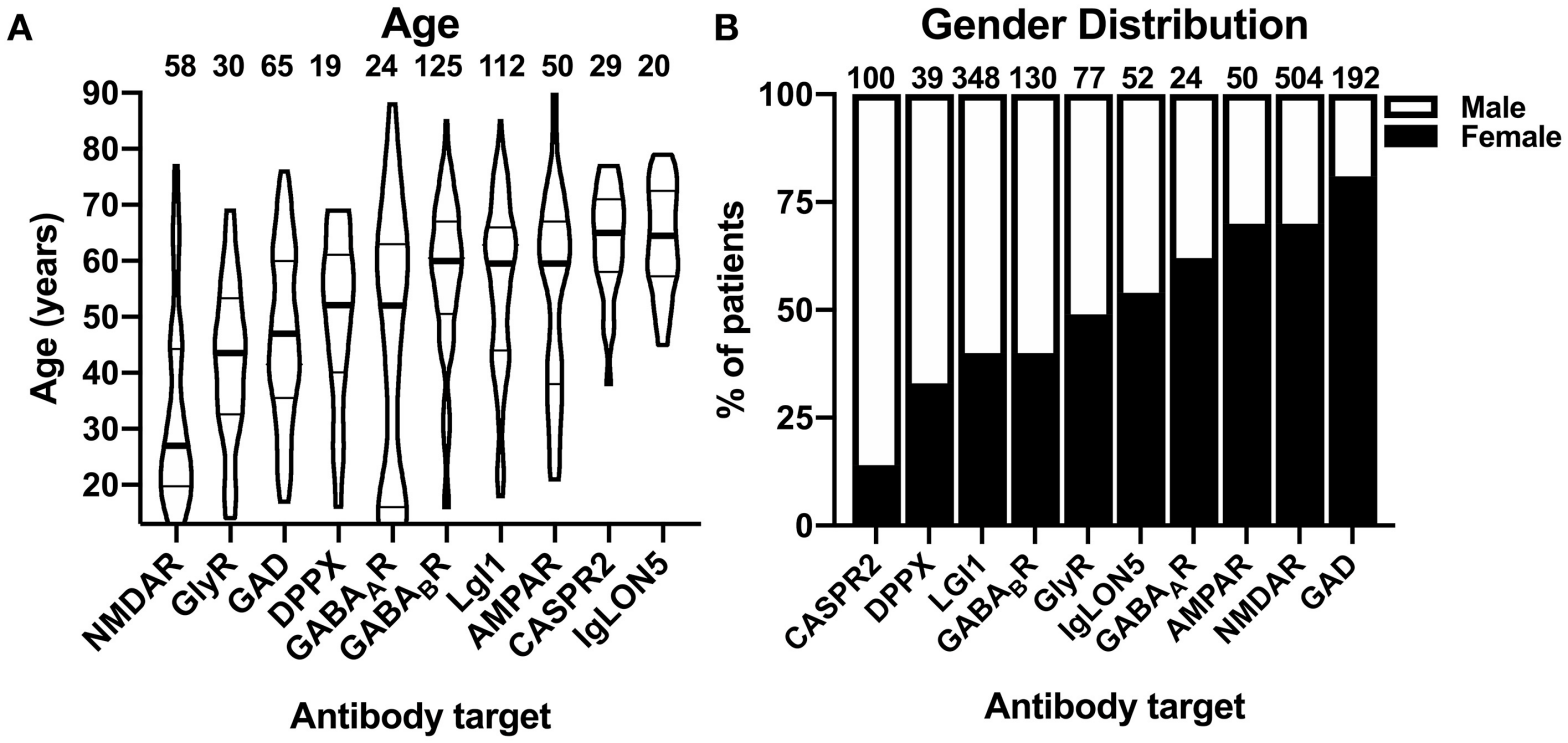
Demographic characteristics of patients with autoimmune encephalitis subtypes defined by 10 different antibodies. **(A)** Distribution of individual reported ages in patients with autoimmune encephalitides (AIE) of the antibody-defined subtypes. The distribution is depicted as violin plots. Patients younger than 12 years were excluded. The median age is indicated as bold line, the interquartile range is indicated by fine lines. Note the bimodal age distribution of the patients with GABA_A_R antibodies. **(B)** Gender distribution of the combined groups of patients with group wise data about the gender distribution available. The percentage of females is depicted as the black part of the bar.

When comparing the basic demographic variables, age, and gender, among the different cohorts with antibody-defined AIE subtypes prominent differences became apparent. Whereas, the median age was 60 years or higher for patients with AIE associated with GABA_B_R, IgLON5, LGI1, CASPR2, and AMPAR antibodies, patients with GABA_A_R, DPPX, GAD, and GlyR antibody-associated AIE were considerably younger (Figure 2A). Patients with GABA_A_R antibodies showed a bimodal age distribution with an additional group of patients with a very young age. Patients with NMDAR antibody-associated AIE were the youngest with a median age of 27 years (Figure 2A). In addition to age, also the gender distributions were very different among the different AIE subtypes. While females were exceedingly rare among patients with CASPR2 antibodies (14%) and males among patients with GAD antibodies (19%), there was a moderate male pre-dominance in AIE with DPPX, LGI1, and GABA_B_R antibodies and a moderate female pre-dominance in AMPAR, GABA_A_R, and NMDAR antibody-positive patients (Figure 2B).

**Figure 3:**
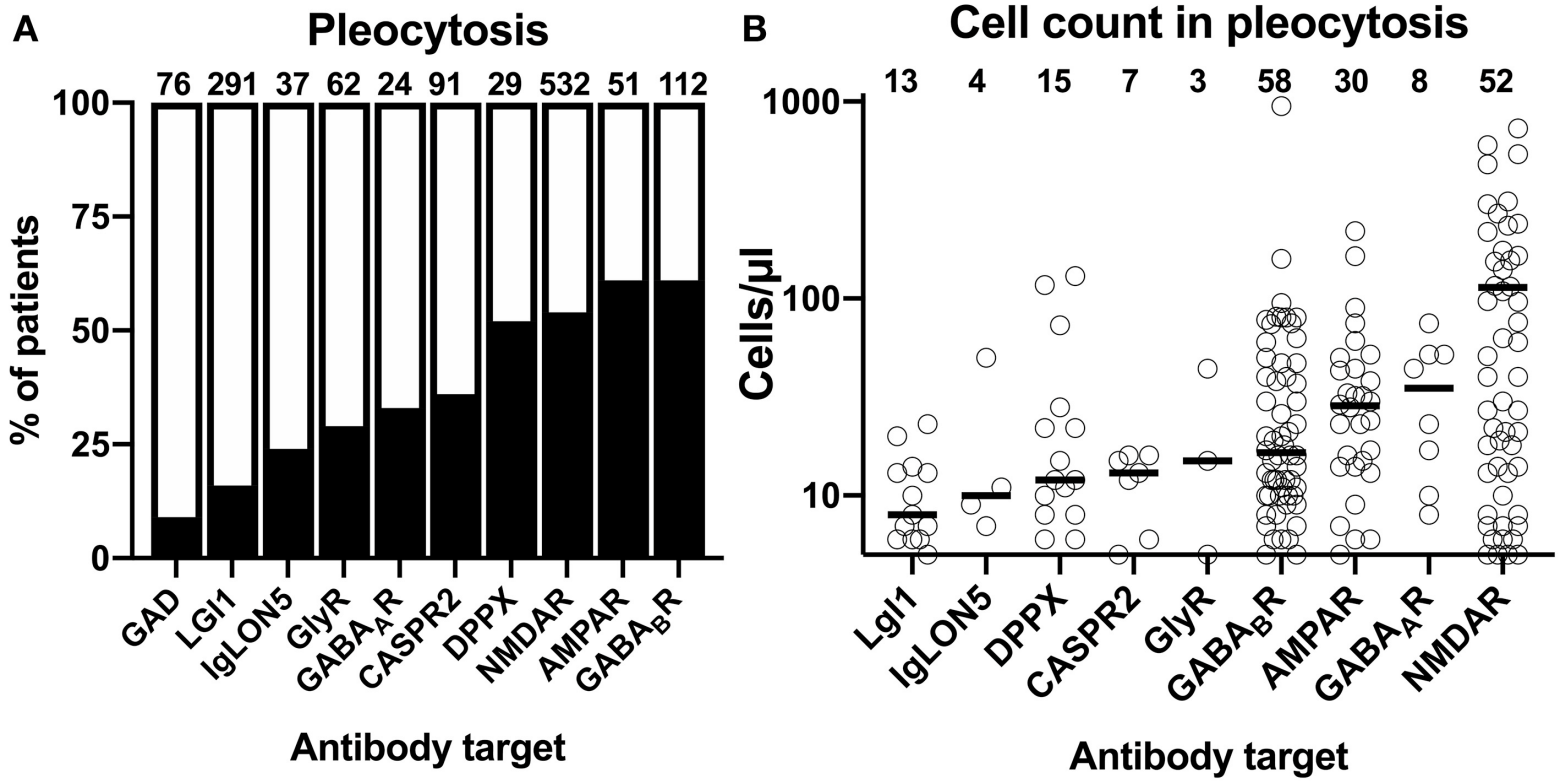
Differential frequency of CSF pleocytosis and CSF cell count in patients with different antibody-defined autoimmune encephalitis subtypes. **(A)** The cumulative percentage of the patient groups identified in the literature with CSF pleocytosis. The percentage of patients with reported CSF pleocytosis is indicted as black. The total number of patients is indicated above each bar. **(B)** Distribution of reported CSF cell counts in individual patients with antibody-defined autoimmune encephalitis (AIE) subtypes and pleocytosis. The median cell count identified as a bold line. The origin of the y-axis is set to 5 cells/μl, the lowest pathological cell count.

The cumulative reported frequencies of CSF pleocytosis were also highly divergent among the 10 different antibody-defined AIE subtypes: while present in 50% or more of the patients with NMDAR, AMPAR, GABA_B_R, and DPPX antibodies, CSF pleocytosis was rare in patients with GAD, LGI1, and IgLON5 antibodies with frequencies of 9, 16, and 24%, respectively (Figure 3A). Patients with the remaining antibodies, anti-GlyR, -GABA_A_R, and -CASPR2, had reported frequencies of CSF pleocytosis ranging from 29 to 36%. Analyzing individual exact CSF cell counts if pathological only, reduced the number of data points considerably. Due to the scarcity of data points, GAD antibodies–only reportedly elevated in two patients with 7 and 56 cells/μl–were omitted from graphical depiction of this analysis (Figure 3B). Median values for CSF pleocytosis of 20 cells/μl and higher were found in AIE associated with AMPAR and NMDAR antibodies as well as for GABA_A_R antibody-associated AIE. The latter findings are surprising, as pleocytosis in these subtypes was found to be rather infrequent (Figure 3A). However, the number of data points were limited with three and eight for GlyR and GABA_A_R antibodies, respectively. A relevant pleocytosis of 20 cells/μl or more was reported for >60% of patients with GABA_B_R, AMPAR, and NMDAR antibodies, corresponding to those with highest percentage of reported pleocytosis, whereas CSF cell counts in this range were found in 40% of patients with DPPX antibodies and finally in 25% or less in patients with AIE associated with IgLON5, LGI1 as well as CASPR2 antibodies, the three subtypes where CSF pleocytosis was least common. Pleocytosis of >100 cells/μl was found in 2 of 58 patients (3%) with GABA_B_R antibodies and in 2 of 30 patients (7%) with AMPAR antibodies, 2 of 15 patients with DPPX antibodies (13%) and 18 of 52 patients (35%) with NMDAR antibodies but not in the other AIE subtypes. Maximal cell counts well above 500 cell/μl were reported for patients with GABA_B_R (950 cell/μl) and NMDAR antibodies (730 cells/μl) only. However, for GABA_B_R antibody-positive AIE this cell counts can be judged as exceptionally high as the next highest cell count was considerably lower (159 cells/μl).

**Figure 4:**
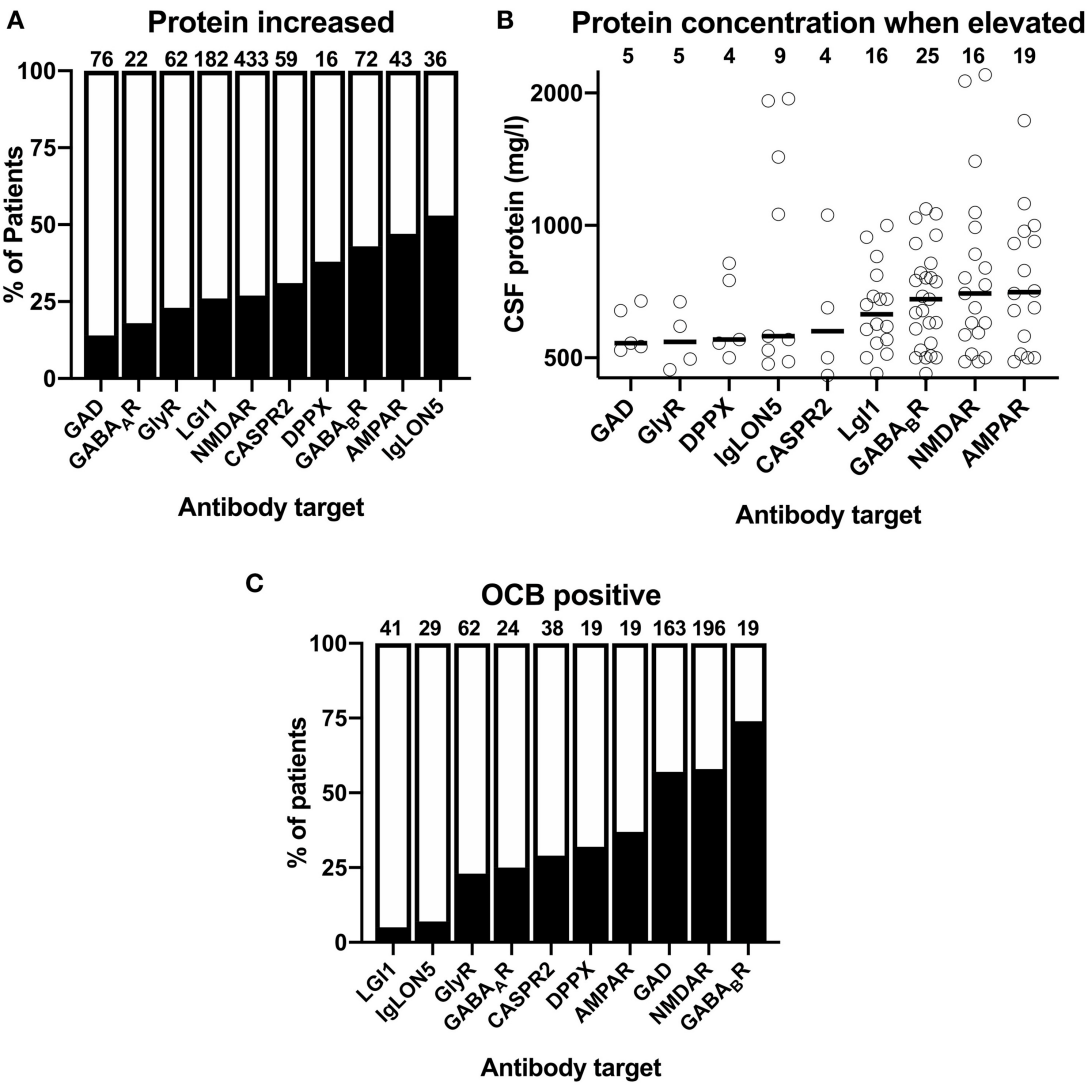
Reported frequencies of increased protein and oligoclonal IgG in the CSF of patients with different antibody-defined autoimmune encephalitis subtypes. **(A)** The cumulative percentage of the antibody-defined AIE patient groups identified in the literature with increased CSF protein. The percentage of patients with reported CSF protein is indicated as black. The total number of patients is indicated above each bar. **(B)** Distribution of reported pathological CSF protein values in individual patients with antibody-defined autoimmune encephalitis (AIE) subtypes. The median cell count identified by a bold line. The origin of the y-axis is set to 450 mg/l as upper normal limit for CSF protein. **(C)** The cumulative percentage of the antibody-defined AIE patient groups identified in the literature with isolated oligoclonal bands (OCB) in the CSF. The percentage of patients with positive OCB is indicated as black. The total number of patients is indicated above each bar.

An elevated CSF protein reportedly occurred in <25% in AIE patients with GAD, GABA_A_R as well as GlyR (Figure 4A). In contrast, in AIE patients with antibodies against AMPAR and GABA_B_R elevated CSF protein levels was reported with a frequency of 43 and 47%, respectively. With reportedly elevated CSF protein in 53% of patients, AIE with IgLON5 antibodies showed in highest prevalence of this finding. Again, the frequencies of elevated individual CSF protein values (>450 mg/l) among cases with individual exact values reported were substantially higher when these were compared to group data (Supplementary Table 2). As for the CSF cell count, we thus omitted all individual patients with normal exact CSF protein levels to avoid that this reporting bias distorts our analysis. For AIE with GABA_A_R antibodies, only two CSF protein values (520 or 600 mg/l) were available. We thus omitted their graphical depiction as these two values were judged as not representative (Figure 4B). When ranking the combined individual pathological protein levels according to their median, two of the AIE subtypes with the highest percentage of reportedly increased in protein, AIE associated GABA_B_R and AMPAR antibodies, were among the four subtypes with the highest median pathological CSF protein levels (Figure 4B). In addition, protein levels reported for patient with LGI1 and NMDAR antibodies, although increased protein was reported much less often, also showed relatively high median pathological protein levels. When analyzed for the frequency of pathological protein levels >1,000 mg/l, these were detected in four of nine patients (44%) with IgLON5, 1 or 4 patients (25%) CASPR2, 4 of 18 patients (22%) with NMDAR, and 4 of 25 patients (16%) with GABA_B_R antibodies.

The third CSF finding we extracted from the published data was the reported presence or absence of isolated OCB in the CSF. In more than 50% of patients with GAD, GABA_B_R, and NMDAR antibodies, positive OCB in the CSF were reported (Figure 4C). With 37%, the frequency of positive OCB was considerably lower in patients with AMPAR antibodies. Between 23 and 32% of patients with antibodies against GlyR, GABA_A_R, CASPR2, and DPPX antibodies were OCB-positive, while positive OCBs were exceedingly rare in the groups of patients with LGI1 and IgLON5 antibodies with a percentage of only 5 and 7%, respectively.

**Figure 5:**
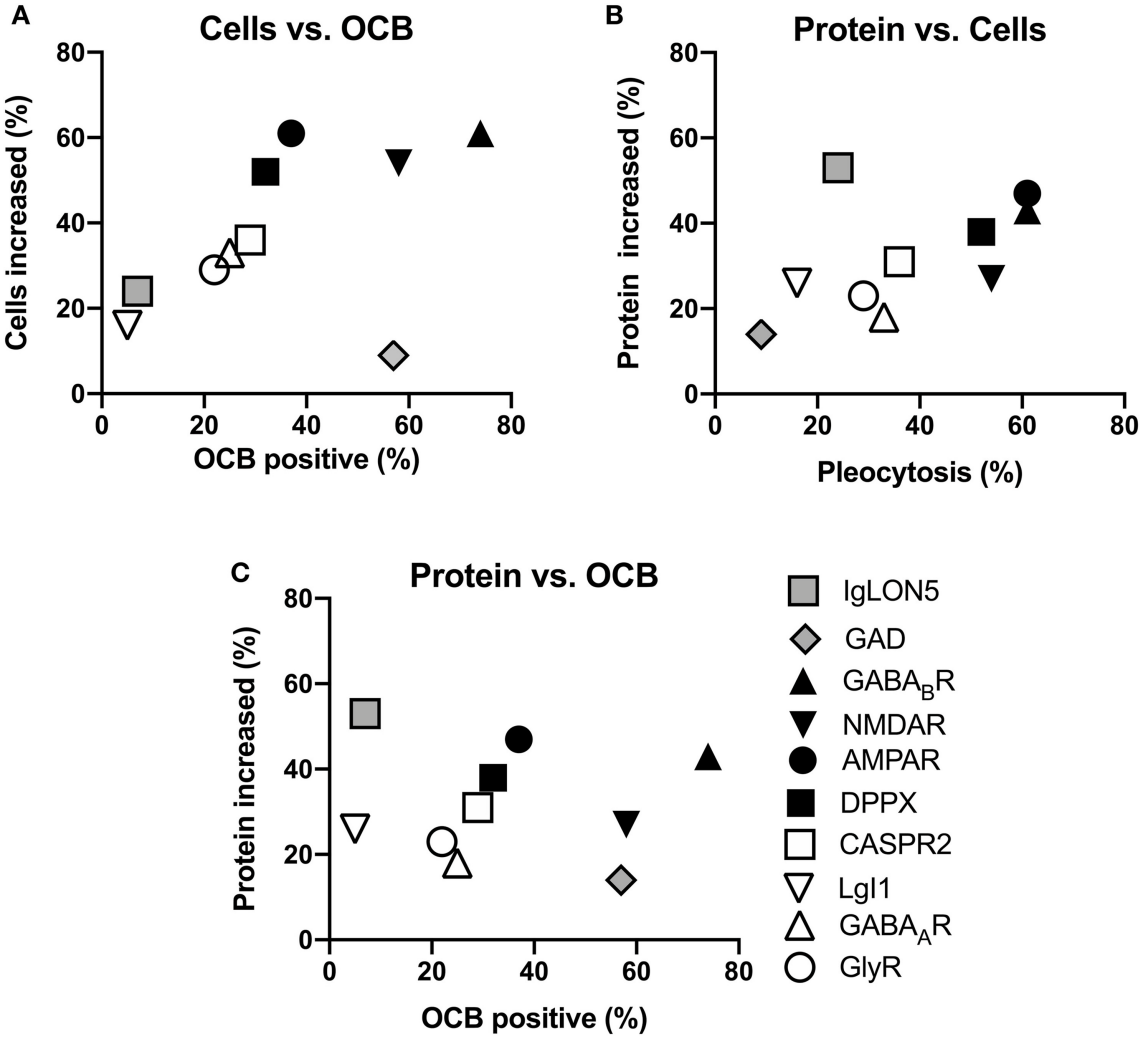
Relationships of the reported frequencies of pleocytosis, elevated protein, and presence of oligoclonal IgG in the CSF of patients with different antibody-defined autoimmune encephalitis subtypes. Graphs depict the cumulative frequencies of reported increased CSF cell counts plotted against the frequencies of reportedly positive oligoclonal IgG [OCB positive, **(A)**], increased CSF protein against pleocytosis **(B)** as well as the frequency of increased protein plotted against OCB positivity **(C)**. The antibody-defined autoimmune encephalitis (AIE) subtypes were grouped as those with infrequent pathological changes (empty symbol: LGI1, GABA_A_R, GlyR, CASPR2), frequent pathological changes (black symbols: AMPAR, DPPX, GABA_B_R, NMDAR) as well as those rather distinct patterns of CSF pathologies (gray symbols: IgLON5, GAD). Compared to the rare occurrence of CSF pleocytosis and positive OCB, CSF of patients with IgLON5 antibodies were frequently reported to exhibit elevated CSF protein. CSF findings in patients with GAD antibodies are characterized by a high frequency of positive OCB while pleocytosis and elevated CSF protein rarely occur.

In summary, the reported percentages of pathological values for the three basic CSF analyses are highly different among the 10 antibody-defined subtypes of AIE examined. However, when the percentage of reported pleocytosis was plotted against the percentage of reportedly positive OCB, it became apparent that subtypes with frequent pleocytosis are in general also characterized by frequently positive OCB (Figure 5A). There was one exception however, as GAD antibody-associated CNS diseases only rarely show CSF pleocytosis while this subtype ranked among high with respect to OCB positivity. When the frequency of reportedly elevated CSF protein was plotted against the frequency of pleocytosis, a similar relationship became apparent. However, here the frequency of elevated CSF protein seemed to be disproportionately high in patients with IgLON5 antibodies (Figure 5B). Similar observations were made when the frequency of elevated CSF protein was plotted against relative OCB positivity (Figure 5C). It seems that AIE subtypes with antibodies against either NMDAR, GABA_B_R, AMPAR, or DPPX are characterized by rather frequent pathological changes in all three analyses, while these CSF abnormalities seem to be rather infrequent in those subtypes with either CASPR2, LGI1, GlyR, or GABA_A_R antibodies. GAD and IgLON5 antibody-positive AIEs deviate from this general pattern with either disproportionally frequent positive OCB or elevated protein levels, respectively.

**Figure 6:**
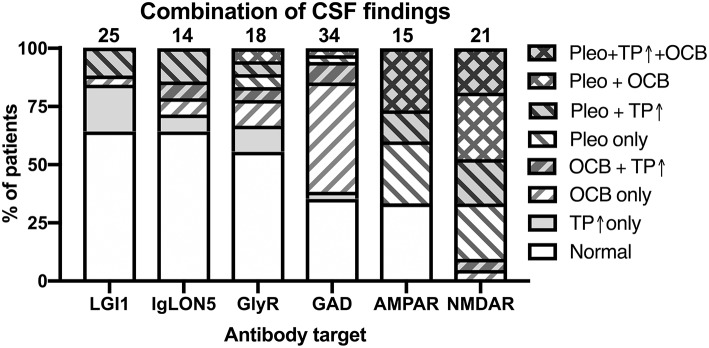
Combination of pathological CSF findings in patients with antibody-defined autoimmune encephalitis. Patients with individual data with regard to all three basic CSF analyses–presence of pleocytosis, elevated CSF protein and positive OCBs–were analyzed for the frequencies of the eight possible combinations of all the tree pathologies. The order of the different antibodies was determined by the percentage of patients with normal or not definitely inflammatory CSF findings (increased protein only). Pleo, pleocytosis; TP ↑, total protein increased; OCB, positive isolated oligoclonal bands in CSF. Diagonal stripes upwards from left to right: positive OCB, downwards: pleocytosis.

Having thus established that the reported data about single CSF findings seem to share certain patterns, we next investigated the relative co-occurrences of pleocytosis, elevated CSF protein, and positive OCB in individual patients suffering from 1 of the 10 antibody-defined AIE subtypes. For that purpose, we expanded our literature search to publications with <3 patients for AIE associated with AMPAR, CASPR2, DPPX, GABA_B_R, GAB_A_AR, and IgLON5 antibodies as for these <15 patients were identified with the full data set. Thereby, we increased the number of patients by 10 for AIE with IgLON5 antibodies (74, 75, 79–86), three for AIE with AMPAR antibodies (8, 10, 11), by two for AIE with DPPX antibodies (35, 38), by one for AIE with CASPR2 (21) and GABA_B_R antibodies each (47). However, the number of individual patients with information of all three CSF parameters remained <10 for AIE with DPPX, CASPR2, GABA_B_R, and GABA_A_R antibodies (Supplementary Table 3). Although the scarcity of data prohibited a more detailed analysis for these four antibodies, cases with CASPR2 antibodies had mostly normal CSF while inflammatory changes were observed in the majority of the other three subtypes. The analysis of the remaining AIE subtypes showed that basic CSF results were normal or unspecifically pathological with elevated CSF protein only in more than 2/3 of patients with LGI1, IgLON5, and GlyR antibodies, while only ~1/3 were normal or unspecifically pathological in AIE with GAD and AMPAR antibodies. In contrast, all patients with NMDAR antibodies had definitively inflammatory CSF findings (Figure 6). In AIE with AMPAR and NMDAR antibodies, the vast majority of patients with inflammatory CSF had pleocytosis with or without positive OCB. However, there was a substantial proportion of patients with GAD antibodies with positive OCB in the absence of pleocytosis (56%), a combination also present in 4, 14, and 17% of patients with LGI1, IgLON5, or GlyR antibodies, respectively.

## Discussion

In clinical neurology, the differential diagnosis of AIE is often considered in patients presenting with new-onset epilepsy, psychiatric diseases, especially in younger patients, and dementia or delirium in the elderly. The rationale is that early identification of AIE might lead to a favorable response to immunosuppressive therapy (2) and missing the diagnosis might lead to life-long cognitive deficits. It is thus not surprising that proving the inflammatory origin of neurological sequelae by CSF findings plays a role in the diagnostic criteria for AIE recently proposed by multiple experts in this field (3). However, it is known that CSF in AIE sometimes lacks inflammatory changes (90, 119).

Within the last years, it became apparent that the different antibody-defined subtypes of AIEs actually represent different diseases with typical clinical presentations, subtype-specific typical ages of onset and gender prevalences as well as imaging results (1). In addition, genome-wide linkage studies demonstrated that fundamentally different genetic risk factors (120). For the two most frequent AIE subtypes, those associated with LGI1 or NMDAR antibodies (6, 121), it is already well-acknowledged that AIE with LGI1 antibodies is rarely associated with inflammatory changes (5, 6, 90) while pleocytosis and/or positive OCB occur in most cases of AIE with NMDAR antibodies (4, 103).

Thus, it is conceivable that each antibody-defined AIE subtype has characteristic CSF findings that reflect its immune pathophysiology. To generate data that support this hypothesis, we performed a systematic evaluation of the CSF findings in published cases with 10 different types of AIE associated with well-defined antineuronal antibodies. In total, we combined the results of 1,305 patients for the presence of pleocytosis, while information about the CSF protein and especially OCB was less often available (Table 1). In addition, only in a minority of patients with individual results, data for all the three CSF parameters were reported. The fact that the exact CSF cell count or CSF protein level were more likely to be reported when abnormal introduced prominent bias and led us to analyze pathological CSF cell count and protein values only to prevent a distortion of our analysis due to differential reporting of normal values among the 10 AIE subtypes. Moreover, the normal values for cell count and CSF protein, as probably the techniques for the determination, slightly differed. In addition, in none of the reports CSF erythrocyte count, which may artificially increase CSF cell count, was reported. Finally, information about the time point of the reported CSF analysis with regard to disease onset or potential immunosuppressive medication administered beforehand was not available in the vast majority of patients. Thus, our results have to interpreted with caution.

Our group analysis indicates that in addition to AIE with NMDAR antibodies also the much rarer AIE subtypes with GABA_B_R and AMPAR and maybe DPPX antibodies not only frequently show CSF pleocytosis but also positive OCB. In contrast, diseases associated with LGI1, IgLON5, CASPR2, and GlyR antibodies rarely show positive OCB as well as pleocytosis. Of note, in these AIE subtypes cell counts when pleocytosis is present, with exception of GlyR, where only a limited number of patients was published, are relatively low compared to AIEs with NMDAR, AMPAR, and GABA_B_R antibodies. These findings were corroborated by our analysis of the typical patterns of individual patients with all three parameters. Thus, it can be expected that AIEs with NMDAR, AMPAR, GABA_B_R, and DPPX antibodies in most cases will show inflammatory CSF changes supporting the diagnosis of an AIE before the results of a specific antibody testing are available, but this will not be the case in most patients with LGI1, IgLON5, CASPR2, and GlyR antibodies. Of note, of the two basal CSF findings to unequivocally prove an inflammatory process, CSF pleocytosis and isolated OCB in the CSF, currently only pleocytosis is included as a criterium for the diagnostic category of possible AIE and definitive limbic encephalitis of autoimmune origin, while for category of possible NMDAR encephalitides both pleocytosis and OCB are considered (3). Correspondingly, with few exceptions in patients with NMDAR-antibody associated AIE with OCB only, pleocytosis was always detected when CSF was OCB-positive in AIE with AMPAR and NMDAR antibodies. However, isolated OCB without pleocytosis were present in patients with LGI1, IgLON5, GlyR, and most prominently with GAD antibodies. Thus, we are not convinced that OCB positivity should be weighted differently than pleocytosis in the diagnostic work-up of suspected AIE. Although, it was estimated that positive OCB occur with a frequency <5% in the healthy individuals (122) and thus their specificity for an active and symptomatic inflammatory process is <100%, the same can be assumed for mild CSF pleocytosis as slightly increased CSF cell counts nowadays might be a consequence of an automated cell count, which frequently overestimates low CSF cell counts (123), although pleocytosis is exceptionally rare in patients with neurodegenerative disease when CSF cell are counted manually (124). In addition, both parameters cross-validate each other when positive.

In general, in AIE subtypes with frequent definitively inflammatory CSF changes, pleocytosis, and/or OCB, increased CSF protein levels reportedly also occurred more frequently and vice versa. Of note, there are two notable exceptions of this rule. Firstly, GAD antibody-associated diseases prominently show a disproportionately high frequency of positive OCB, while CSF pleocytosis or elevated protein are exceptionally rare. Secondly, patients with IgLON5 antibodies might be characterized by rather frequent and high elevations of CSF protein in the absence of pleocytosis and positive OCB. Of note, IgLON5 antibody-associated encephalopathy differs from both AIE subtypes with rare and frequent inflammatory CSF changes by its poor response to immunosuppression (73).

In our analysis, CASPR2 antibody-associated AIE showed an intermediate to low frequency of pleocytosis and OCB. Of note, for this AIE, different subtypes have been described: limbic encephalitis associated with high CASPR2 antibodies in CSF, Morvan's syndrome (MoS) with low anti-Caspr2 antibodies in serum only, and finally cerebellar ataxia (23, 27). However, CSF findings of MoS are not included in our CASPR2 antibody-positive cohort, as these were without exception reported grouped with either CSF findings of LGI1 antibody-positive patients (125) or patients with peripheral hyperexcitability only (23).

GABA_A_R antibodies have been reported in a variety of neurological diseases, even in patients finally diagnosed to suffer from a hereditary disease (39, 42). However, a specific AIE subtype presenting as a rather acute and severe encephalopathic syndrome with multifocal T2 hyperintensities upon MRI as well as severe epilepsy is characterized by detection of anti-GABA_A_R antibodies in both serum and CSF (40). We focused our analysis on patients with positive CSF antibodies. Although, the number of patients was limited, our analysis indicates that although pleocytosis is only observed in a minority of patients, cell counts frequently exceed of >20 cells/μl when elevated. Further studies have to investigate the clinical relevance of this finding.

Autoimmune encephalitides (AIE) associated with NMDAR antibodies is the most frequent AIE subtype (121), preferentially occurs at younger age (4) and, in our analysis, is almost always associated with inflammatory CSF changes, while AIE associated with LGI1 antibodies, which might be the second most common form with an annual incidence of more that 1:1 million (6) and typically occurs at older age and in males (5), rarely shows inflammatory CSF changes. Taken together, this strongly supports the hypothesis that inflammatory CSF changes might have a much higher discriminatory power to tell AIE from schizophrenia within the second or third decade of life, especially in females, than delirium or rapid progressive dementia from AIE in late life, especially in males. Of note, our findings underscore a recent report of antibody-associated neurological syndromes without signs of inflammation in the elderly (119).

The assumption that different immunological processes underlie the AIE subtypes is corroborated by the pre-dominant IgG subclasses reportedly involved (1). While the most prevalent antigen-specific IgG subclass is IgG4 in AIEs associated with IgLON5 (73), CASPR2 (24), LGI1 (126) an DPPX antibodies (1), in AIEs with GABA_B_R, NDMAR, and AMPAR antibodies these were classified as pre-dominantly IgG1 (1). However, in line with IgG1-dependent complement fixation in AIE with LGI1 antibodies (127, 128), this subclass might be additionally important in this AIE subtype (126). AIEs with AMPAR and NMDAR antibodies, in our analysis with highly similar CSF findings, also share the pathogenic mechanism, receptor internalization rather than complement fixation (129–131).

AIE subtypes reportedly characterized by antigen-specific IgG1 reportedly are more likely to be paraneoplastic than those where antigen-specific IgG4 prevails (1). Combining these categorizations with the results of our analysis allows the hypothesis that a pathophysiology more likely to be paraneoplastic and driven by antigen-specific antibodies of the IgG1 subclass might be associated with robust and frequent inflammatory CSF findings, while non-paraneoplastic AIE subtypes with IgG4 as pre-dominant antigen-specific antibody rarely show an inflammatory CSF. GAD antibody-associated AIEs do not fit into this scheme. However, GAD is an intracellular antigen and thus cytotoxic T cells might play a prominent role in GAD antibody-associated AIE (127), as demonstrated for diabetes type 1 associated with GAD antibodies (132), a related and often co-existing disease. GAD antibody-associated CNS diseases are characterized by a much more chronic course compared to the other AIE subtypes (57). Correspondingly, we show that the typical pattern of CSF changes in GAD antibody-associated CNS disease is very different from all other AIE subtypes due to the pre-dominance of positive OCB while pleocytosis and increased CSF protein are rare.

In summary, our findings suggest that different antibody-defined AIE subtypes are associated with characteristic CSF findings. Rather non-paraneoplastic and IgG4 pre-dominant disease subtypes tend to have less CSF inflammatory activity compared to diseases with IgG1 pre-dominance, which more frequently are paraneoplastic. AIE with NMDAR antibodies is the most frequent AIE subtype at younger age and almost always associated with inflammatory CSF findings while anti-LGI1 AIE, the most frequent AIE subtype in the elderly, in the majority of patients CSF is normal. We thus conclude that in suspected AIE in the elderly, normal basic CSF findings should not lead to the decision against testing for antineuronal antibodies. As this assumption is based on a retrospective review of the literature, they have to be confirmed prospectively diagnosed patients.

## Data Availability

Publicly available datasets were analyzed in this study. This data can be found here: https://www.ncbi.nlm.nih.gov/pubmed/.

## Author Contributions

TB performed the literature research, extracted the data and did the analysis, and critically revised the manuscript. JL envisioned the concept of the analysis, supervised data acquisition, and wrote the manuscript.

### Conflict of Interest Statement

JL leads a laboratory where detection of the antibodies to diagnose autoimmune encephalitis is performed. He is a member of the scientific advisory board of the German Network for Research on Autoimmune encephalitides (GENERATE) and member of the executive board of the German Society for Cerebrospinal Fluid Diagnostics and Clinical Neurochemistry. He received funding from the BMBF for a project have covers the topic of autoimmune encephalitides. The remaining author declares that the research was conducted in the absence of any commercial or financial relationships that could be construed as a potential conflict of interest.
