# Supplementary material for: Cerebrospinal Fluid Findings in Patients With Autoimmune Encephalitis—A Systematic Analysis
Source: Front Neurol. 2019 Jul 25;10:804. doi: 10.3389/fneur.2019.00804 (PMC6670288; doi:10.3389/fneur.2019.00804)
Supplement: Supplementary file 1 [file Data_Sheet_1.docx]

| **Antibody target** | **Ratio number of patients with individual values vs. group data** | | | |
| --- | --- | --- | --- | --- |
|  | **Gender** | **Cells** | **TP** | **OCB** |
| **AMPAR** | **100 %** | **67 %** | 42 % | 84 % |
| **CASPR2** | 22 % | 19 % | 14 % | 21 % |
| **DPPX** | 49 % | 52 % | 31 % | 95 % |
| **GABA_A_R** | **100 %** | 42 % | 9 % | **100 %** |
| **GABA_B_R** | 95 % | 64 % | **64 %** | 95 % |
| **GAD** | 34 % | 8 % | 16 % | 40 % |
| **GlyR** | 42 % | 18 % | 18 % | 29 % |
| **IgLON5** | 38 % | 11 % | 25 % | 55 % |
| **LGI1** | 32 % | 10 % | 14 % | 80 % |
| **NMDAR** | 12 % | 12 % | 10 % | 18 % |

**Supplementary Table 1:** Proportion of the number of patients with individual data available compared to the number of patients with group data in the publications identified by the literature search. The **highest** percentages are indicated by a dark grey background and the lowest by clear background

| **Antibody target** | **% Females**  **(group/single exact values)** | **% Pleocytosis**  **(group/single exact values)** | **% TP↑**  **(group/single exact values)** | **% OCB+**  **(group/single)** |
| --- | --- | --- | --- | --- |
| **AMPAR** | **70/70** | 61/91** | 47/89** | 37/25 |
| **CASPR2** | 14/23 | 36/44 | 31/50 | 29/13 |
| **DPPX** | 33/26 | 52/100** | 38/100* | 32/33 |
| **GABA_A_R** | 63/63 | 33/80* | 18/100 | **25/25** |
| **GABA_B_R** | 40/38 | 61/72 | 43/54 | 74/72 |
| **GAD** | 81/80 | 9/33 | 14/42* | 57/66 |
| **GlyR** | 49/56 | 29/27 | 23/36 | 22/22 |
| **IgLON5** | 54/45 | 24/100** | 53/100** | 7/13 |
| **LGI1** | 40/43 | 16/43*** | 26/64*** | 5/6 |
| **NMDAR** | 70/65 | 54/81**** | 27/44* | 58/71 |

**Supplementary Table 2:** Analysis whether the groups of patients with individual exact data for AIEs with specific antibodies are representative for the total group only published with group data. TP↑ = total protein increased, OCB+ = positive oligoclonal IgG restricted to the CSF. Dark grey: identical, light grey: non-significantly different, clear background: significantly higher. Statistical analysis was done be Fisher’s exact test: *p<0.05, **p<0.01, ***p<0-001, ****p<0.0001.

|  | Normal | TP | Pleo | Pleo+  TP | OCB | TP+  OCB | Pleo+  OCB | Pleo+TP  +OCB | N |
| --- | --- | --- | --- | --- | --- | --- | --- | --- | --- |
| AMPAR | 5(33) | 0(0) | 4(27) | 2(13) | 0(0) | 0(0) | 0(0) | 4(27) | 15 |
| CASPR2 | 4(67**)** | 0(0) | 0(0) | 1(17) | 1(17) | 0(0) | 0(0) | 0(0) | 6 |
| DPPX | 1(20) | 0(0) | 1(20) | 2(40) | 0(0) | 0(0) | 0(0) | 1(20) | 5 |
| GABA_A_R | 0(0) | 0(0) | 4(50) | 1(13) | 0(0) | 0(0) | 1(13) | 2(25) | 8 |
| GABA_B_R | 1(13) | 1(13) | 2(25) | 0(0) | 0(0) | 1(13) | 2(25) | 1(13) | 8 |
| GAD | 12(35) | 1(3) | 1(3) | 0(0) | 16(47) | 3(9) | 1(3) | 0(0) | 34 |
| GlyR | 10(56) | 2(11) | 1(6) | 1(6) | 2(11) | 1(6) | 1(6) | 0(0) | 18 |
| IgLON5 | 9(64) | 1(7) | 0(0) | 2(14) | 1(7) | 1(7) | 0(0) | 0(0) | 14 |
| LGI1 | 16(64) | 5(20) | 0(0) | 3(12) | 1(4) | 0(0) | 0(0) | 0(0) | 25 |
| NMDAR | 0(0) | 0(0) | 5(24) | 4(19) | 1(5) | 1(5) | 6(29) | 4(19) | 21 |

**Supplementary Table 3:** Distribution of different combinations of CSF pathologies among the different antibody-defined AIE subtypes. The values correspond to Figure 6 with the exception that antibody-defined AIE subtypes for which less than ten subjects with all three parameters reported as either normal or pathological are not included in Figure 6 (grey background).
